# Evaluation of the effect of gluten‐free diet and Mediterranean diet on autoimmune system in patients with Hashimoto's thyroiditis

**DOI:** 10.1002/fsn3.3833

**Published:** 2023-11-20

**Authors:** Mutlu Tuçe Ülker, Gözde Arıtıcı Çolak, Murat Baş, Mustafa Genco Erdem

**Affiliations:** ^1^ Department of Nutrition and Dietetics, Faculty of Health Sciences Istı̇nye University Istanbul Turkey; ^2^ Health Sciences Institute Acıbadem Mehmet Alı̇ Aydınlar University Istanbul Turkey; ^3^ Department of Nutrition and Dietetics, Faculty of Health Sciences Acıbadem Mehmet Alı̇ Aydınlar University Istanbul Turkey; ^4^ Department of Internal Medicine, Faculty of Medicine Beykent University Istanbul Turkey; ^5^ Istinye University Gaziosmanpasa Medicalpark Hospital Istinye University Istanbul Turkey

**Keywords:** gluten‐free diet, Hashimoto, Mediterranean diet

## Abstract

Hashimoto's thyroiditis is an autoimmune disease in which thyroid cells are attacked through cell‐and antibody‐mediated immune processes. A gluten‐free diet reduces antibody concentration and regulates thyroid autoimmunization. Mediterranean diet reduces oxidative stress. This study evaluates the short‐term effects of Mediterranean, gluten‐free, and Mediterranean gluten‐free dietary patterns on thyroid function and autoantibody levels of patients. The 40 patients with Hashimoto's thyroiditis included in the study were randomly divided into four groups (defined as gluten‐free, Mediterranean, Mediterranean gluten‐free, and controls) for 12 weeks. Thyroid function tests, autoantibody levels, and food consumption were recorded at the beginning and end of the study. There was no statistically significant difference in TSH levels of the groups before the intervention, but a statistically significant difference was found afterward (*p* < 0.05). Free T_3_ hormone levels showed a statistically significant difference across the groups before and after the intervention (*p* < 0.05). Free T_3_ hormone levels increased significantly in all intervention groups after the intervention, with the highest increase in the Mediterranean group (*p* < 0.05). In the intervention groups, anti‐TPO and anti‐Tg levels decreased after the intervention; however, this difference was not significant across groups (*p* > 0.05). In addition, body weight, body mass index, waist and hip circumference averages decreased significantly in all intervention groups compared with controls (*p* < 0.05). The study achieved an increase in Free T_3_ hormone levels in the intervention groups. The most marked difference was seen in the Mediterranean gluten‐free diet model, which may be due to the anti‐inflammatory effect of both Mediterranean and gluten‐free diets and the loss of body weight as a result of the intervention.

## Graphical abstract

This is the first study to evaluate the effect of multiple dietary patterns on thyroid function, body composition, nutrient intake, and nutritional knowledge in Hashimato thyroid patients.

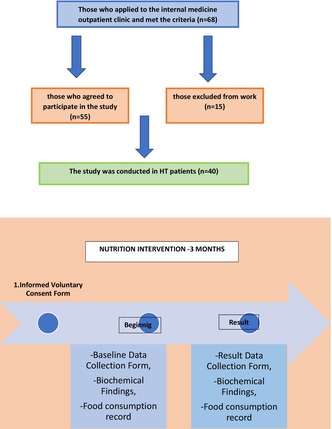

## 1 INTRODUCTION

Hashimoto's thyroiditis (HT) is an autoimmune disease characterized by the production of thyroid autoantibodies against thyroid peroxidase (TPO) and thyroglobulin (Tg) that attack thyroid cells by cell‐ and antibody‐mediated immune processes and cause progressive fibrosis (Ragusa et al., 2019). Thyroid hormones are responsible for thermogenesis, basal metabolic rate, carbohydrate, protein and fat metabolism, so HT adversely affects health status and quality of life. Thyroid peroxidase plays an important role in the synthesis of thyroid hormones such as free thyroxine (FT_4_) and free triiodothyronine (FT_3_). Tg is a glycoprotein that acts as a source of thyroid hormones (Chahardoli et al., 2019).

Although the etiology of HT is not known exactly, it is thought to be related to the interaction between genetic factors, environmental factors, and epigenetic effects and is 7–10 times more common in women than in men. Since cellular and humoral immunity play a role in the development of the disease, inflammatory infiltration of T and B cells may be seen. The diagnosis of HT is based on anti‐thyroid antibodies and histological features (Ralli et al., 2020). Numerous autoimmune environmental triggers of HT include sedentary lifestyle, changes in dietary habits, and psychological stress. Western‐style diet, consumption of processed foods, high‐fat, high‐sugar, high‐salt, and low‐fiber diets also increase the risk of HT by altering the composition of the microbiota (Zheng et al., 2023). Increased animal fat intake increase the risk of developing anti‐TPO and anti‐Tg antibodies, which may be reduced by a diet rich in vegetables, fruits, and nuts. Recently, iodine, selenium, and gluten intake have gained importance in the dietary plans of HT patients (Kaličanin et al., 2020). Iodine is an essential microelement in the diet for the synthesis of FT_3_ and FT_4_ and the maintenance of thyroid gland function. Selenium is an essential mineral in the homeostasis of several critical functions related to the immune system and signal transduction pathways and is involved in the structure of antioxidant enzymes (Mikulska et al., 2022).

Hashimoto's thyroiditis diet therapy is based on proper nutrition of the body and regulation of the immune system with an anti‐inflammatory diet. Intake of adequate protein and dietary fiber, and the n‐3 family of unsaturated fatty acids, especially n‐3, are important. In HT patients, interactions of gliadin with thyroid antigens are often observed (Ihnatowicz et al., 2020). Gluten‐free diet (GFD) helps regulate thyroid autoimmunization by reducing the concentration of tissue transglutaminase antibodies (Pobłocki et al., 2021). It has been reported that omega‐3 fatty acids in the Mediterranean diet (MD) in particular play a role in the regulation of inflammatory responses, reduce inflammation. Due to the anti‐inflammatory effects of the MD, disease‐induced oxidative stress parameters have been found to be lower in HT patients, which may reduce inflammation in thyroid tissue (Osowiecka & Myszkowska‐Ryciak, 2023). The Mediterranean gluten‐free diet (MGFD) is a combination of GFD and MD and recommends the consumption of water, fresh fruit, vegetables, and cereals, as well as GF cereals, so it may be effective against inflammation seen in HT (Bascuñán et al., 2020).

To our knowledge, the effect of different diets affects thyroid autoimmunity and function in HT patients has not been compared in previous studies. This study evaluates the short‐term effects of MD, GFD, and MGFD on autoimmune systems in HT patients.

## 2 MATERIALS AND METHODS

### 2.1 Patient population

The study participants were selected from newly diagnosed and untreated female HT patients aged 18–65 years who applied to the internal medicine outpatient clinic between June 2021 and May 2022. To be accepted for the study, the following characteristics were required: (a) positive anti‐TPO (>5.61 IU/mL) and positive anti‐Tg (>4.11 IU/mL), (b) thyroid function tests (TSH, FT_3,_ and FT_4_), (d) decreased echogenicity of the thyroid parenchyma on thyroid ultrasonography; (e) no symptoms of celiac disease and no diagnosis; (f) GFD diet in the last 3 months; and (g) Body Mass Index (BMI) between 18.5 kg/m^2^ and 30 kg/m^2^. The study excluded women on thyroid function medication, women following an autoimmune diet, women with other diagnosed autoimmune diseases, and pregnant or breastfeeding women.

The research was conducted in accordance with the Declaration of Helsinki. The Medical Research Evaluation Board of Acıbadem Mehmet Ali Aydınlar University granted medical ethics approval for the study (decision number 2021–09/17 dated 26.05.2021). All participants gave their informed consent for the study.

### 2.2 Study design

This study is a prospective, single‐blind study including case and control groups. Before the study, participants were informed about its benefits and risks. Participants were randomly divided into four groups (MD, GFD, MGFD, and controls). The intervention groups (MD group (*n* = 10), GFD (*n* = 10), MGFD (*n* = 10)) were administered weekly diets by a dietitian for 12 weeks according to individual requirements and daily energy needs; weight loss was not targeted. Patients in controls (*n* = 10) did not receive any special dietary intervention. Patients were interviewed weekly to assess adherence to the study protocol, and food consumption was recorded at each interview; those who complied with the weekly planned menus by less than 80 percent were excluded from the study.

### 2.3 Thyroid function of patients

Thyroid function tests (TSH, FT_4_, FT_3_, anti‐TPO and anti‐Tg) ordered by the physician at the beginning and at the end of the study (3 months later) were measured by enzyme‐linked immunosorbent assay (ELISA) in the hospital laboratory and obtained from patient files with the permission of the hospital (detection sensitivity of the kits; anti‐Tg: <4.11 IU/mL; anti‐TPO: <5.61 IU/mL; TSH: 0.35–4.94 uIU/mL; FT_3_: 1.58–3.91 pg/mL and FT_4_: 0.7–1.48 ng/dL).

### 2.4 Anthropometric measurements

Anthropometric measurements (height, body weight, and waist–hip circumference) were taken at the beginning and end of the study. Tanita Bc‐601 bioelectrical impedance analysis (BIA) weighing device was used for body weight and analysis. A portable stadiometer, with a non‐flexible tape measure, was used to record height and waist and hip circumferences. All measurements were taken in accordance with the International Standards for Anthropometric Assessment (ISAK) guidelines, wearing light clothing and no shoes (Isak—The International Society for the Advancement of Kinanthropometry). BMI, waist–height and waist–hip ratios of the participants were calculated by the researcher and evaluated according to World Health Organization parameters (World Health Organization (WHO), 2006).

### 2.5 Evaluation of food intake

We recorded three‐day food consumption (2 days on weekdays and 1 day on weekends) at the beginning and end of the study, and a photographic food catalog was used to portion sizes (Keskinkılınç & Yardım, 2017). Data obtained from food consumption records and food consumption frequencies were analyzed with the BEBIS version 9 program (Beslenme Bilgi Sistemi (BeBİS 9), 2019).

### 2.6 Statistical analysis

The sample size of the study was calculated using the G*Power 3.1.9 program. The a priori hypotheses were anti‐TPO, GFD dietary pattern before and after the intervention, type I error *α*:0.05, effect size as large effect, targeted power of the test 1‐*β*:0.80 and sample size n:10 and a total of 40 patients were included. All data were analyzed on computer using SPSS (statistical package for social sciences for Windows 22) program. Kruskal–Wallis‐H test was used to compare more than two independent groups and Bonferroni test, one of the post hoc tests, was used to determine the source of the difference when significant. Wilcoxon test was used to analyze the difference between dependent numerical variables. Chi‐squared test and Fisher's exact test were used to the difference between categorical independent variables and McNemar and McNemar–Bowker test were used to analyze dependent categorical variables, *p* < 0.05 was deemed significant.

## 3 RESULTS

**FIGURE 1:**
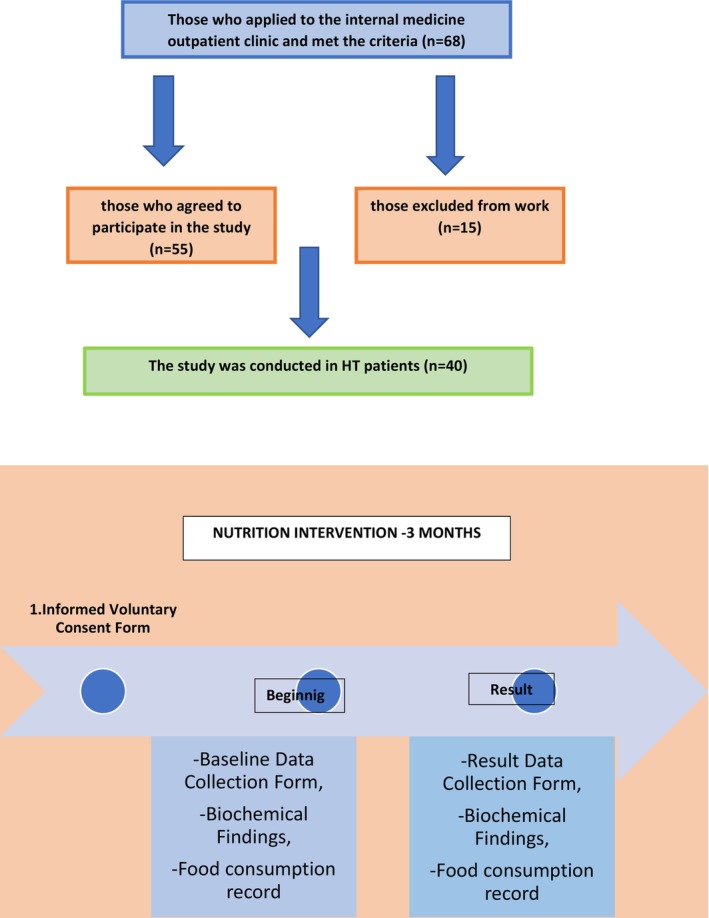
Flowchart of the research.

Sixty‐eight patients with HT met the study inclusion criteria, but only 55 of the candidates voluntarily agreed to participate in the study. Fifteen HT patients did not comply with the nutritional intervention and were excluded from the study, and the study was completed with 40 HT patients, Figure 1 summarizes the flow time of the study, and Table 1 presents basic characteristics of the patients.

**TABLE 1:** Distribution of demographic characteristics of patients.

	*n*	%
Education level		
Secondary school	2	5.0
High school	8	20.0
University	30	75.0
Age (years)		
25–35	15	37.5
36–45	18	45.0
45+	7	17.5
$\bar{X}$ ± SD (Min‐Max)	39.05 ± 7.52 (25–57)	

*Note*: %, percentage; *n*, number.

### 3.1 Participant thyroid function

While TSH levels (μIU/mL) did not show a statistically significant difference across the groups before the intervention, a significant difference was found before the intervention, a significant difference was found across the groups after the intervention (*p* < 0.05). According to Bonferroni multiple comparison test, TSH levels in the GFD group were lower than controls, TSH levels in all intervention groups increased and were within the reference range. FT_3_ (pg/mL) hormone levels showed a statistically significant difference across the groups before and after the intervention (*p* < 0.05). FT_3_ hormone levels increased significantly in all intervention groups after the intervention, with the highest increase in the MD group (*p* < 0.05), post‐intervention FT_3_ hormone levels were lower in the GFD group compared with the control and MGFD groups. FT_4_ (ng/dL) hormone levels did not show a significant difference across the groups before and after the intervention (*p* > 0.05). There was a statistically significant difference across the anti‐TPO (IU/mL) and anti‐Tg (IU/mL) levels anti‐TPO and anti‐Tg levels decreased in these groups after the intervention and decreased more in the MD and MGFD groups (*p* < 0.05); however, this difference was not significant across the groups (Table 2).

**TABLE 2:** Thyroid function tests, baseline, and 3 months of post‐intervention.

	Group	Before intervention	After intervention	Test value/*p*	
		Median (min‐max)	Median (min‐max)	*z*	*p*
TSH (uIU/mL)	Control group	2.51 (1.99–4.60)	2.56 (2.46–4.40)	−1.07	.28
	MD	1.75 (1.25–2.76)	2.10 (1.21–2.83)	−0.76	.44
	GFD	1.26 (1.05–1.76)	1.47 (1.26–1.90)	−1.17	.24
	MGFD	1.65 (0.93–3.71)	1.92 (0.96–3.97)	−1.78	.07
		*X* ^2^ = 5.20 *p* = .16	*X* ^2^ = 8.32 *p* = .04 Difference = 3 < 1		
FT_3_ (pg/mL)	Control group	2.99 (2.80–3.87)	3.03 (2.90–3.88)	−1.24	.21
	MD	2.50 (2.45–2.70)	2.86 (2.78–2.94)	−2.80	.01
	GFD	2.60 (2.46–2.67)	2.71 (2.61–2.93)	−2.19	.03
	MGFD	3.30 (2.98–3.50)	3.57 (3.21–3.67)	−2.80	.01
		*X* ^2^ = 18.76 *p* = .00 Difference = 2.3 < 1.4	*X* ^2^ = 12.96 *p* = .00 Difference = 3 < 1.4		
FT_4_ (ng/dL)	Control group	14.06 (0.95–15.80)	14.50 (0.98–16.60)	−2.70	.01
	MD	0.91 (0.90–0.94)	0.97 (0.93–0.98)	−2.44	.01
	GFD	0.97 (0.88–1.04)	0.99 (0.98–1.28)	−2.09	.04
	MGFD	1.01 (0.94–1.30)	1.25 (0.98–1.54)	−2.80	.01
		*X* ^2^ = 7,11 *p* = .07	*X* ^2^ = 8.80 *p* = .03		
Anti TPO (IU/mL)	Control group	61.05 (36.11–76.67)	59.55 (30.67–76.90)	−1.27	.20
	MD	70.76 (47.88–102.30)	68.95 (46.32–90.80)	−2.80	.01
	GFD	150.29 (44.68–267.55)	133.25 (42.41–247.30)	−2.70	.01
	MGFD	257.56 (90.30–386.31)	140.20 (82.40–320.10)	−2.80	.01
		*X* ^2^ = 6.97 *p* = .07	*X* ^2^ = 5.53 *p* = .14		
Anti Tg (IU/mL)	Control group	22.85 (21.44–23.30)	21.65 (19.80–31.20)	−0.05	.96
	MD	20.00 (8.96–52.37)	13.74 (6.89–50.80)	−2.80	.01
	GFD	7.50 (2.05–56.70)	6.31 (2.03–40.47)	−2.80	.01
	MGFD	32.66 (19.87–46.50)	25.77 (15.60–43.20)	−2.80	.01
		*X* ^2^ = 4.32 *p* = .23	*X* ^2^ = 3.50 *p* = .32		

Abbreviations: GFD,Gluten‐free diet; MD, Mediterranean diet; MGFD, Mediterranean gluten‐free diet; *X* ^2^, Ki square, Krukal Wallis‐*H*; *z*,Wilcoxon test.

### 3.2 Participant anthropometric measurements

There was a statistically significant difference in body weight (kg), BMI (kg/m^2^), and waist and hip circumference (cm) before and after the intervention in MD, GFD, and MGFD groups (*p* < 0.05). Body weight, BMI, waist and hip circumference averages decreased significantly in all intervention groups compared with controls (*p* < 0.05). After the intervention, the difference in the values of body weight, BMI, and hip circumference was greatest in the MD group, and the difference in the value of waist circumference was greatest in the MGFD group. Body fat percentage did not show a statistically significant difference across the groups before and after the intervention (*p* > 0.05). Body muscle percentage difference across the groups was statistically after the intervention (*p* < 0.05), and body muscle percentage in the controls was lower than in the MD, GFD, and MGFD groups (Table 3).

**TABLE 3:** Anthropometric measurements, baseline, and 3 months of post‐intervention.

	Group	Before intervention	After intervention	Test value/*p*	
		Median (min‐max)	Median (min‐max)	*z*	*p*
Body Weight (kg)	Control group	57.50 (49.00–76.00)	56.70 (49.00–75.00)	−0.06	.95
	MD	75.70 (63.50–88.00)	67.50 (57.00–85.70)	−2.80	.01
	GFD	59.10 (58.10–73.40)	55.90 (54.05–65.70)	−2.80	.01
	MGFD	69.15 (63.00–76.00)	64.05 (57.90–72.90)	−2.80	.01
		*X* ^2^ = 6.79, *p* = .08	*X* ^2^ = 3.41, *p* = .33		
Body Mass Index (kg/m^2^)	Control group	22.34 (20.40–28.60)	22.15 (20.32–28.23)	−0.17	.86
	MD	28.55 (25.39–35.70)	25.64 (22.60–32.49)	−2.80	.01
	GFD	22.92 (22.50–23.43)	21.57 (20.12–22.27)	–2.80	.01
	MGFD	23.22 (21.80–27.25)	22.01 (20.31–26.14)	–2.80	.01
		*X* ^2^ = 8.67, *p* = .03 Difference = 1 < 2	*X* ^2^ = 6.32, *p* = .10		
Waist Circumference (cm)	Control group	88.50 (87.00–102.00)	88.00 (87.00–105.00)	−0.73	.46
	MD	93.00 (80.00–103.00)	90.50 (76.00–96.00)	−2.81	.00
	GFD	78.50 (72.00–88.00)	77.00 (71.00–85.00)	−2.82	.00
	MGFD	89.00 (73.00–95.00)	85.00 (70.00–92.00)	−2.84	.00
		*X* ^2^ = 6.75, *p* = .08	*X* ^2^ = 8.12, *p* = .04 Difference = 3 < 1		
Hip Circumference (cm)	Control group	95.50 (92.00–102.00)	95.50 (91.00–102.00)	−0.93	.35
	MD	111.00 (100.00–124.00)	108.50 (98.00–119.00)	−2.82	.00
	GFD	96.00 (94.00–99.00)	94.00 (92.00–94.00)	−2.82	.00
	MGFD	97.00 (94.00–110.00)	94.00 (92.00–107.00)	−2.85	.00
		*X* ^2^ = 10.50, *p* = .01 Difference = 1 < 2 and 3 < 4	*X* ^2^ = 10.02, *p* = .02 Difference = 3 < 2		
Body Muscle Percentage (%)	Control group	37.35 (29.00‐56.55)	27.85 (26.30–30.50)	−1.68	.09
	MD	54.38 (32.90–66.37)	50.96 (32.40–66.41)	−0.53	.59
	GFD	55.19 (52.40–68.21)	56.20 (44.90–56.80)	−0.15	.88
	MGFD	47.75 (45.60–66.37)	49.45 (44.30–68.90)	−1.98	.05
		*X* ^2^ = 4.64, *p* = .20	*X* ^2^ = 14.64, *p* = .00 Difference = 1 < 2.3.4		
Body Fat Percentage (%)	Control group	34.05 (28.70‐39.70)	32.90 (27.70–37.80)	−0.20	.84
	MD	37.20 (30.20‐43.20)	37.40 (25.20–40.10)	−1.88	.06
	GFD	29.25 (26.70‐32.10)	28.20 (24.90–30.70)	−2.59	.01
	MGFD	31.85 (27.60‐36.70)	29.65 (25.80–33.40)	−2.80	.01
	*X* ^2^ = 4.17, *p* = .24		*X* ^2^ = 4.55, *p* = .21		

Abbreviations: GFD,Gluten‐free diet; MD, Mediterranean diet; MGFD, Mediterranean gluten‐free diet; *X* ^2^, Ki square, Krukal Wallis‐*H*; *z*,Wilcoxon test.

### 3.3 Participant food intake

There was no statistically significant across the groups in daily energy intake before and after the intervention. Carbohydrate, protein, and fat intake (%) were not statistically significantly different across the groups before the intervention and after the intervention, protein intake was higher in the MD group than in the GFD and MGFD groups, and carbohydrate and fat intake was lower in the MD group than in the GFD and MGFD groups (*p* < 0.05) (Table 4).

**TABLE 4:** Daily dietary energy, macronutrient, and micronutrient intakes of patients at baseline and after 3 months of intervention.

	Group	Before intervention	After intervention	Test value/*p*	*p*
		Median (min‐max)	Median (min‐max)	*z*	
Energy (kcal)	Control group	1677.85 (1253.80–1926.80)	1715.55 (1565.70–1829.70)	−0.41	.68
	MD	1308.15 (1253.80–1800.20)	1717.90 (1441.40–1937.50)	−1.58	.11
	GFD	1610.65 (1315.10–1792.90)	1713.20 (1614.30–1790.00)	−0.88	.37
	MGFD	1753.05 (1441.40−1829.70)	1812.30 (1591.40–1926.80)	−1.27	.20
		*X* ^2^ = 2.63 *p* = .45	*X* ^2^ = 1.30 *p* = .73		
Protein (%)	Control group	20.00 (19.00–22.00)	21.00 (19.00‐22.00)	−0.23	.81
	MD	17.00 (15.00–22.00)	25.00 (22.00–26.00)	−2.67	.01
	GFD	20.00 (16.00–22.00)	20.00 (19.00–20.00)	−0.42	.67
	MGFD	20.00 (19.00–21.00)	19.50 (15.00–20.00)	−1.31	.19
		*X* ^2^ = 0.88 *p* = .83	*X* ^2^ = 19.92 *p* = .00 Difference = 2 > 3.4		
Carbohydrate (%)	Control group	66.85 (63.50–81.80)	81.30 (63.50–89.00)	−1.00	.31
	MD	60.40 (58.50–64.40)	60.10 (24.60–70.50)	−0.66	.51
	GFD	74.40 (64.40–79.00)	83.60 (64.00–84.60)	−0.53	.59
	MGFD	80.45 (60.10–90.00)	85.40 (79.00–92.20)	−1.37	.17
		*X* ^2^ = 5.58 *p* = .13	*X* ^2^ = 16.37 *p* = .00 difference = 2 < 3.4		
Fat (%)	Control group	39.50 (36.00‐44.00)	39.00 (36.00–43.00)	−0.05	.95
	MD	41.00 (33.00–44.00)	19.00 (16.00–31.00)	−2.80	.01
	GFD	41.50 (38.00–46.00)	42.00 (36.00–43.00)	−1.25	.21
	MGFD	39.50 (33.00–43.00)	39.00 (38.00–53.00)	−1.12	.26
		*X* ^2^ = 2.83 *p* = .42	*X* ^2^ = 21.49 *p* = .00 Difference = 2 < 1.3.4		

Abbreviations: GFD,Gluten‐free diet; MD, Mediterranean diet; MGFD, Mediterranean gluten‐free diet; *X* ^2^, Ki square, Krukal Wallis‐*H*; *z*,Wilcoxon test.

## 4 DISCUSSION

The most important finding of the study was the increase in FT_3_ hormone levels in HT patients in the intervention groups, especially in the MD group. There was also a decrease in autoantibody levels in the intervention groups compared with pre‐intervention; however, this difference was not significant across the groups. This suggests that GFD and MD, an anti‐inflammatory diet, are effective against inflammation in HT. In addition, the highest difference in the anthropometric findings of the patients was seen in the MD group. Given the detailed and strict inclusion and exclusion criteria in HT patients, the results of our study cannot be attributed to comorbid disorders or the effect of any medication taken by patients, the post‐intervention changes are the result of individualized planned nutritional interventions.

A study by Zupo et al. evaluating the effect of MD on thyroid function found a significant negative correlation (*p* < 0.05) across compliance with MD and FT_3_ hormone and FT_4_ hormone levels (Zupo et al., 2020). A study by Krysiak et al. after GFD was administered to HT patients for 3 months, FT_3_ hormone levels of the patients increased compared with control (Krysiak et al., 2019). In our study, while the FT_3_ hormone level in the MD and GFD groups was significantly lower than the control and MGFD groups at the beginning, the GFD group was significantly lower than the control and MGFD groups after the intervention (*p* = 0.00). This statistically significant (*p* = 0.01) difference is due to the change in FT_3_ hormone level in the Mediterranean group. A is attributed to be due to the fact that increased intake of fruits and vegetables, selenium, zinc, folic acid, and antioxidants in the composition of the MD model affects inflammation and thus autoimmunity in HT (Ruggeri et al., 2021).

In a study conducted by Pobłocki et al. evaluating the effect of GFD on thyroid function and autoantibodies in HT patients for 12 months, TSH levels decreased significantly in the intervention group than in controls; however, no significant difference was found in anti‐TPO and anti‐Tg antibodies, FT_3_ or FT_4_ levels (*p* > 0.05) (Pobłocki et al., 2021). In a study conducted by Krysiak et al. in which 34 female patients with HT were administered GFD for 6 months, no significant change was found in the TSH levels of patients in the GFD group compared with controls (*p* > 0.05) (Krysiak et al., 2019). In our study, there was no significant difference across the groups in TSH levels before the intervention. At the end of the study, the TSH level of the gluten‐free group was significantly lower than controls (*p* = 0.04), the TSH levels of the patients increased, however, they were still in the reference range, this result may be related to the effectiveness of nutritional interventions.

Thyroid peroxidase is a key enzyme in the synthesis of T_3_ and T_4_ hormones. In HT, thyroid autoantibodies against TPO and Tg are produced and FT_4_ hormone levels of patients decrease. At the beginning of the study, there was no significant difference across the groups in terms of FT_4_ hormone levels (*p* > 0.05). This was a criterion in the randomization of the patients, and this result may be related to the new diagnosis of the patients. At the end of GFD administered to HT patients for 12 months by Metso et al., no significant difference was found in FT_4_ hormone levels of patients in the intervention group compared with controls, but anti‐TPO and anti‐Tg levels decreased (Metso et al., 2012). In our study, the increase in FT_4_ hormone levels was higher and significant in the MD group compared with controls (*p* = 0.01); however, the difference was not statistically significant when FT_4_ hormone levels were evaluated across the groups (*p* > 0.05).

Anti‐TPO is a good indicator of thyroid autoimmunity and thyroid gland damage increases as anti‐TPO level increases (Szczuko et al., 2022). In a meta‐analysis of 22 studies (Song et al., 2019), obesity was found to have a statistically significant association with anti‐TPO autoantibody levels (*p* = 0.001); however, no significant association was found with anti‐Tg levels. At the beginning of our study, there was no significant difference in anti‐TPO levels across the groups (*p* > 0.05), anti‐TPO levels were above the reference value, and patients were randomized so that there was no difference across anti‐TPO positivity and anti‐TPO levels. In our study, similar to the results of the meta‐analysis study, body weight, BMI values, and anti‐TPO and anti‐Tg levels of the patients decreased significantly; however, this was not significant across groups, obese patients were not included in the study to evaluate the effectiveness of the intervention better because of the need for medication and treatment caused by metabolic disorders.

Ostowska et al found that in HT patients, after 3 months of GFD, body weight, BMI, body fat percentage, TSH, anti‐TPO levels, and FT_4_ hormone levels of the intervention group decreased significantly compared with controls (Ostrowska et al., 2021). Our study found patients' BMI, body weight, body fat percentage, and anti‐TPO levels decreased and TSH and FT_4_ hormone levels increased after different nutritional interventions. Although there was no energy restriction in our study, the decrease in body weight and thyroid autoantibody levels in all intervention groups suggests that the intervention was effective; however, the highest difference was in the MGFD group, which may be due to the anti‐inflammatory effect of MD and GFD and the loss of body weight as a result of the intervention. There are no studies in the literature evaluating the effects of different nutritional interventions on BMI and body weight in HT patients, and the studies in the literature are based on the comparison of a nutritional model with a control group; however, in this study, BMI and body weight of individuals were evaluated after more than one nutritional intervention.

Since gluten‐containing foods such as barley, wheat, and rye are restricted in gluten‐restricted diets, individuals' carbohydrate intake may be reduced or gluten‐free foods made from them (El Khoury et al., 2018).While there was no difference in carbohydrate intake across the groups before the intervention, carbohydrate intake of the MD group was significantly higher than all groups after the intervention (*p* = 0.00). This may be due to restriction of cereal intake due to gluten elimination in GFD groups.

An anti‐inflammatory diet rich in vitamins A, C, E, polyphenols, omega‐3 fatty acids and minerals such as magnesium, copper, magnesium, zinc and selenium may be more effective than eliminating gluten in reducing thyroid autoantibody levels (Ihnatowicz, Drywien, et al., 2020). In this study, anti‐TPO and anti‐Tg levels of the patients decreased after nutritional intervention compared with the pre‐intervention period; however, there was no significant difference across the groups. FT3 levels of the patients increased. This study evaluates the short‐term effect of nutritional interventions applied to HT patients, and different studies are needed for the long‐term evaluation of hormone and antibody levels in patients undergoing nutritional intervention. It is thought that body weight loss may also be effective on these results.

### 4.1 Importance and contribution of the study

In our study, the effects of different nutritional interventions on thyroid function tests and thyroid antibodies were evaluated and an increase in function tests compared with pre‐intervention and a decrease in antibody levels compared to pre‐intervention were found; however, the most important finding was the increase in FT_3_ hormone levels.

### 4.2 Strength of the study

This study is the first to evaluate the effect of multiple dietary patterns on thyroid function, body composition, nutrient intake, and nutritional knowledge in HT patients.

### 4.3 Limitations

The study's limitations include the short follow‐up and small number of participants. In addition, because the subjects were all female, the study did not address the effects of nutritional interventions on antibody titers and thyroid function tests in male subjects. The use of non‐thyroid‐specific FFQs to define diet quality is another limitation. Furthermore, the study was conducted under pandemic conditions and the impact of COVID−19 transmission on patients' autoimmune system remains unclear. In all, this study should be considered a pilot study. Prospective studies of longer duration and larger sample size are needed to support its findings.

This study was presented by Mutlu Tuçe Ülker at the thesis defense examination on October, 2022, within the scope of Acıbadem Mehmet Ali Aydınlar University Institute of Health Sciences Nutrition and Dietetics Doctorate Program, but this study has not been presented at any other congress or meeting.

## AUTHOR CONTRIBUTIONS

**Mutlu Tuçe Ülker:** Conceptualization (lead); data curation (lead); formal analysis (lead); investigation (lead); methodology (lead); project administration (equal); resources (lead); software (lead); supervision (lead); validation (lead); visualization (lead); writing – original draft (lead); writing – review and editing (lead). **Gözde Arıtıcı Çolak:** Conceptualization (equal); data curation (lead); methodology (lead); project administration (lead); software (equal); writing – original draft (supporting); writing – review and editing (supporting). **Murat Baş:** Conceptualization (lead); investigation (lead); methodology (lead); project administration (lead); resources (supporting); software (supporting); writing – original draft (lead); writing – review and editing (lead). **Mustafa Genco Erdem:** Conceptualization (lead); methodology (lead); project administration (lead); software (lead); visualization (lead); writing – original draft (lead); writing – review and editing (lead).

## CONFLICT OF INTEREST STATEMENT

The authors declare no conflict of interest.

## Data Availability

Data supporting the findings of this study are available from the authors upon request.
